# Lack of Effects of a Single High-Fat Meal Enriched with Vegetable n-3 or a Combination of Vegetable and Marine n-3 Fatty Acids on Intestinal Peptide Release and Adipokines in Healthy Female Subjects

**DOI:** 10.3389/fnut.2016.00038

**Published:** 2016-08-31

**Authors:** Ingunn Narverud, Mari C. W. Myhrstad, Karl-Heinz Herzig, Toni Karhu, Tuva B. Dahl, Bente Halvorsen, Stine M. Ulven, Kirsten B. Holven

**Affiliations:** ^1^Department of Nutrition, Institute for Basic Medical Sciences, University of Oslo, Oslo, Norway; ^2^Department of Health, Nutrition and Management, Faculty of Health Sciences, Oslo and Akershus University College of Applied Sciences, Oslo, Norway; ^3^Institute of Biomedicine and Biocenter of Oulu, Oulu University Medical School, University of Oulu, Oulu, Finland; ^4^Medical Center Oulu, Oulu University Hospital, Oulu, Finland; ^5^Research Institute of Internal Medicine, Oslo University Hospital, Oslo, Norway; ^6^Faculty of Medicine, University of Oslo, Oslo, Norway; ^7^National Advisory Unit for Familial Hypercholesterolemia, Department of Endocrinology, Morbid Obesity and Preventive Medicine, Oslo University Hospital, Oslo, Norway

**Keywords:** high marine and vegetable fat meal, eicosapentaenoic acid, α-linolenic acid, human, intestinal peptides, adipokines

## Abstract

Peptides released from the small intestine and colon regulate short-term food intake by suppressing appetite and inducing satiety. Intake of marine omega-3 (n-3) fatty acids (FAs) from fish and fish oils is associated with beneficial health effects, whereas the relation between intake of the vegetable n-3 fatty acid α-linolenic acid and diseases is less clear. The aim of the present study was to investigate the postprandial effects of a single high-fat meal enriched with vegetable n-3 or a combination of vegetable and marine n-3 FAs with their different unsaturated fatty acid composition on intestinal peptide release and the adipose tissue. Fourteen healthy lean females consumed three test meals with different fat quality in a fixed order. The test meal consisted of three cakes enriched with coconut fat, linseed oil, and a combination of linseed and cod liver oil. The test days were separated by 2 weeks. Fasting and postprandial blood samples at 3 and 6 h after intake were analyzed. A significant postprandial effect was observed for cholecystokinin, peptide YY, glucose-dependent insulinotropic polypeptide, amylin and insulin, which increased, while leptin decreased postprandially independent of the fat composition in the high-fat meal. In conclusion, in healthy, young, lean females, an intake of a high-fat meal enriched with n-3 FAs from different origin stimulates intestinal peptide release without any difference between the different fat compositions.

## Introduction

Peptides released from the small intestine and colon such as cholecystokinin (CCK), glucose-dependent insulinotropic polypeptide (GIP), and peptide YY (PYY) are regulating short-term food intake by suppressing appetite and inducing satiety. CCK is released in response to nutrients in the duodenal lumen, whereof fat and protein produce a greater postprandial release than carbohydrates (1–3). Lipids significantly stimulate CCK release (4, 5), and the length of the fatty acids (FAs) determines the amount of CCK release (6). FAs with more than 10 carbons in the chain are the most potent stimulants for CCK release (5, 6). However, little is known about the effect of intake of marine versus vegetable omega-3 (n-3) FAs, and whether the release of CCK is influenced differently by these FAs *in vivo*. GIP potentiates meal-induced insulin secretion from pancreas (7), and the release is stimulated by nutrients shortly after ingestion. The major stimuli are dietary fat and carbohydrates (8–10), and fat quality seems to play a role since intake of olive oil has been shown to induce a higher plasma concentration of GIP than butter (11). PYY is released in the intestine, in response to food intake shortly after ingestion (12). The release of PYY is proportional to calorie intake but also the macronutrient composition of the meal affects postprandial PYY release. Dietary fat, carbohydrates, and protein all stimulate PYY release but to different degrees and with different time courses (13). In addition, both chain length and degree of saturation seem to play a role in fat-induced PYY release (14–16). Insulin and amylin are peptides released from the pancreas, which are also affecting short-term food regulation. Glucose is the most powerful regulator of both insulin and amylin (17); however, less is known about the effect of vegetable and marine n-3 FAs on amylin release.

The adipose tissue is constituted by a variety of cells, e.g., adipocytes and macrophages, which secrete adipokines (18). Adipokines are the endocrine mediators of the adipose tissue and include leptin, adiponectin, resistin, and nicotinamid phosphoribosyl transferase (NAMPT)/visfatin (19). Leptin has previously been shown to regulate food intake (20), and ingestion of a high-fat meal or carbohydrate-rich meal has been shown to reduce postprandial levels of adiponectin (21). NAMPT has previously been shown to be suppressed after an oral glucose tolerance test (22); however, little is known about the postprandial effects after ingestion of n-3 enriched high-fat meals on these adipokines. A link between gut peptides and adipocytes has previously been described, and GIP has been shown to enhance insulin-stimulated glucose transport and stimulation of fatty acid synthesis and incorporation into triglycerides (23).

Intake of marine n-3 FAs, eicosapentaenoic acid (EPA; 20:5), and docosahexaenoic acid (DHA; 22:6), from fish and fish oils is associated with beneficial cardiovascular health effects (24, 25). Vegetable oils such as linseed oil and rapeseed oil are the main dietary sources of the vegetable n-3 FA α-linolenic acid (ALA; 18:3). The association between intake of ALA and diseases is less clear since it has been studied less extensively than the marine n-3 FAs (26, 27). Animal studies and studies in humans have suggested that n-3 FAs potentially elicit a number of beneficial health effects including weight reduction *via* suppression of appetite (28). We hypothesized that vegetable n-3 or a combination of vegetable and marine n-3 FAs have a different effect on intestinal peptides and adipokines compared with saturated FAs (SFAs).

The aim of the present study was, therefore, to investigate the postprandial effects of a single high-fat meal enriched with vegetable n-3 or a combination of vegetable and marine n-3 FAs on intestinal peptides and adipokines in lean, healthy females.

## Subjects and Methods

### Subjects

Sixteen healthy, normal weight young women were recruited among students at Akershus University College, Oslo, Norway, in October 2008. Of the 16 females, 2 discontinued after the first test day due to events unrelated to the study, and they were, therefore, not included in the analysis. Three subjects performed only two out of the three test days. This study was conducted according to the guidelines laid down in the Declaration of Helsinki, and all procedures involving human subjects were approved by the Regional Committee of Medical Ethics, south-east region of Norway. Written informed consent was obtained from all subjects.

### Study Design and Test Meal

Study design and test meals have been described in details previously (29). Participant characteristics are given below. Briefly, the participants consumed three different test meals in a fixed order, and all test days were separated by 2 weeks. Postprandial blood samples were taken at 3 and 6 h after the beginning of the test meals (0 h). The three test meals consisted of a 150 g chocolate cake containing the same amount of energy (1923–1977 kJ/100 g) and similar % of energy (E%) from protein (14 E%), total fat (67–70 E%), and carbohydrates (16–19 E%) but contained different fatty acid composition (29). Coconut fat was used as a source for SFA and was the most dominant fat type in all the cakes. The coconut cake contained 43 E% saturated fat and 11 E% polyunsaturated FAs (PUFAs) of which only 1 E% was n-3 FA (ALA; 18:3). In the linseed cake, some of the coconut fat was replaced by fat from linseed oil as a source of vegetable n-3 PUFA where ALA is the primary FA. The linseed cake contained 30 E% of SFA and 22 E% of PUFA, of which n-3 FAs contributed with 14 E% (ALA). In the cod liver cake, some of the coconut fat was replaced by cod liver oil as a source of marine n-3 FAs and linseed oil as a source of vegetable n-3 FAs to give a distinct n-3 PUFA profile from the linseed cake (29). This cake contained 31 E% SFA and 14 E% PUFA of which 10 E% was n-3 FAs (5 E% ALA, 2 E% EPA, and 3 E% DHA). The fatty acid composition of the three different cakes is shown in Table 1.

**Table 1:** **Fatty acid composition of the three different cakes**.

	Coconut cake g/100 g	Linseed cake g/100 g	Cod liver cake g/100 g
C10:0	1.1	0.64	0.61
C12:0	9.4	5.4	5.2
C14:0	3.6	2	2.3
C16:0	4.8	3.7	4
C16:1, n-7	0.1	0.1	0.1
C18:0	4.2	3.8	3.5
C18:1, n-7	0.2	18	0.58
C18:1, n-9	5.1	6.7	5.7
C18:2, n-6	5.4	3.9	1.8
C18:3, n-3	0.6	7.6	2.7
C20:0	0.1	0.1	0.08
C20:1, n-9	<0.1	<0.07	1.3
C20:4, n-6	<0.1	<0.07	0.08
C20:5, n-3	<0.1	<0.07	0.93
C22:1, n-11	<0.1	<0.07	0.7
C22:5, n-3	<0.1	<0.07	0.13
C22:6, n-3	<0.1	<0.07	1.3

### Blood Sampling and Analysis

Blood samples taken from the antecubital veins in heparinized tubes were immediately centrifuged at 4°C, and the plasma stored at −80° until peptide determination. Appropriate lab biosafety procedures were followed when handling the human specimens. Plasma CCK levels were analyzed *via* an radioimmunoassay (RIA) kit (Euro-Diagnostica AB, Malmö, Sweden) after solid phase extraction, as previously described (30, 31). The plasma samples were extracted with a SepPac C18 cartridge preconditioned with 1.5 mL of 2-propanol and 1.5 mL of 0.1% trifluoroacetic acid (TFA; Waters, Milford, AM, USA) in an automated Gilson Aspec XL system (Gilson, Middleton, WI, USA). Plasma samples were acidified with 1M HCl containing 1.6% glycine. After loading the sample, the cartridge was washed with 2 mL of 0.1% TFA, and samples were eluted with 2 mL of 80% acetonitrile in 0.1% TFA. Samples were evaporated into dryness overnight. Dry residue was dissolved in 500 μL of RIA buffer, and RIA was conducted according to manufacturer’s instructions. Briefly, samples were incubated with the primary antibody for 48 h at 4°C and I-125 labeled CCK-8 was added before 96 h incubation at 4°C. Double antibody solid phase was added, and samples were incubated for 60 min at 4°C. Before, the measurement samples were centrifuged 15 min × 1700 *g* in 4°C. The supernatant was decanted, and the activity of aliquot was measured with gammacounter (Wallac 1272 Clinigamma, PerkinElmer, Waltham, MA, USA). The intra-assay variation for the CCK measurement was 3.6%. For CCK measurements, several samples were below detection level and were, therefore, set to the lowest detection level [coconut group *n* = 12, *n* = 3, and *n* = 8; linseed group *n* = 11, *n* = 2, and *n* = 1; cod liver group *n* = 10, *n* = 3, and *n* = 1, for each time point (0, 3, and 6 h), respectively]. PYY, GIP, amylin, and insulin were determined using a Milliplex human gut hormone panel (#HGT-68K, Millipore, MA, USA) and measured in Bio-Plex 200 system based on Luminex xMAP technology (Bio-Rad Laboratories Inc., CA, USA). The kit was performed according to manufacturer’s instructions. The intra-assay and the inter-assay variations were <11 and <19%, respectively. The results were calculated with Bio-PlexManager Software 6.0 with five-parameter logistical equation. The instrument was set for high sensitivity range. Concentration of leptin, total adiponectin, and resistin were measured by enzyme-linked immunosorbant assays (ELISA) from R&D Systems (Minneapolis, MN, USA). Concentration of NAMPT was measured by ELISA from Phoenix Pharmaceuticals (Burlingame, CA, USA). The intra- and inter-assay CVs was <10% for all assays. The analyses were performed in autumn 2010 and spring 2011. The samples had not been defrosted prior to the analyses.

### Peripheral Blood Mononuclear Cells Isolation and mRNA Analysis

After blood collection, PBMCs were isolated using the BD Vacutainer Cell Preparation tubes according to the manufacturer’s instructions (Becton, Dickinson and Company, Franklin Lakes, NJ, USA). Pellets were frozen and stored at −80°C for further RNA isolation.

Total RNA isolation and mRNA analysis were performed as previously described (29). From two subjects in the postprandial study, PBMCs could not be retrieved during the three test days. Therefore, the number of subjects in the gene expression analyses are *n* = 12, *n* = 11, and *n* = 10 for the coconut, linseed, and cod liver cakes, respectively. Briefly, for reverse transcription quantitative polymerase chain reaction (RT-qPCR), we used TaqMan Array Custom Micro Fluidic cards for leptin receptor (Hs00174497_m1, Applied Biosystems, Foster City, CA, USA) and inventoried TaqMan gene expression assay for NAMPT (Hs00237184_m1, Applied Biosystems). The target genes were normalized to the following endogenous controls glucuronidase β (GUSβ) and TATA box-binding protein (TBP) (both TaqMan Array Custom Micro Fluidic cards and inventoried TaqMan gene expression assays cat# Hs99999908_m1 and Hs00427620_m1, respectively, Applied Biosystems). The relative mRNA level for each transcript was calculated by the ΔΔ cycle threshold (Ct) method (32). Briefly, the Ct values for each target gene was normalized against the mean of the Ct values for the two endogenous controls GUSβ and TBP (=ΔCt). ΔΔCt was then calculated as ΔCt at 3 or 6 h after meal intake minus ΔCt at fasting level (0 h). The fold change in mRNA expression was calculated as 2^−ΔΔCt^.

### Statistical Analysis

Each subject consumed three test meals and was used as their own reference. Non-parametric statistics were used throughout the study due to the low number of participants. Data are given as median (interquartile range). The significance of the difference between the meals at 3 and 6 h and between time points for each test meals were assessed with Friedman’s Anova, followed by Wilcoxon matched-pairs test at significant values using exact serum/plasma values (between time points) or delta serum/plasma changes from fasting (between meals), and regarding mRNA fold change from endogenous controls (between time points) or fold change from fasting values (between meals). Probability values (exact, two-tailed) were considered significant at values of *P* < 0.05. All calculations were performed using SPSS (version 19.0). Missing values in the Wilcoxon matched-pairs test analysis were excluded test-by-test.

## Results

### Characteristics of the Participants

The participants in the postprandial study were healthy without infectious diseases, normal weight, young women, with an age of 24 years (22–25 years), body mass index of 22 kg/m^2^ (21–25 kg/m^2^), total cholesterol 4.8 mmol/L (3.6–5.2 mmol/L), fasting triglycerides 0.8 mmol/L (0.7–1.1 mmol/L), and fasting glucose 4.6 mmol/L (4.3–4.9 mmol/L) (29).

### Effects on Plasma Appetite- and Glucose-Regulating Hormones

**Figure 1:**
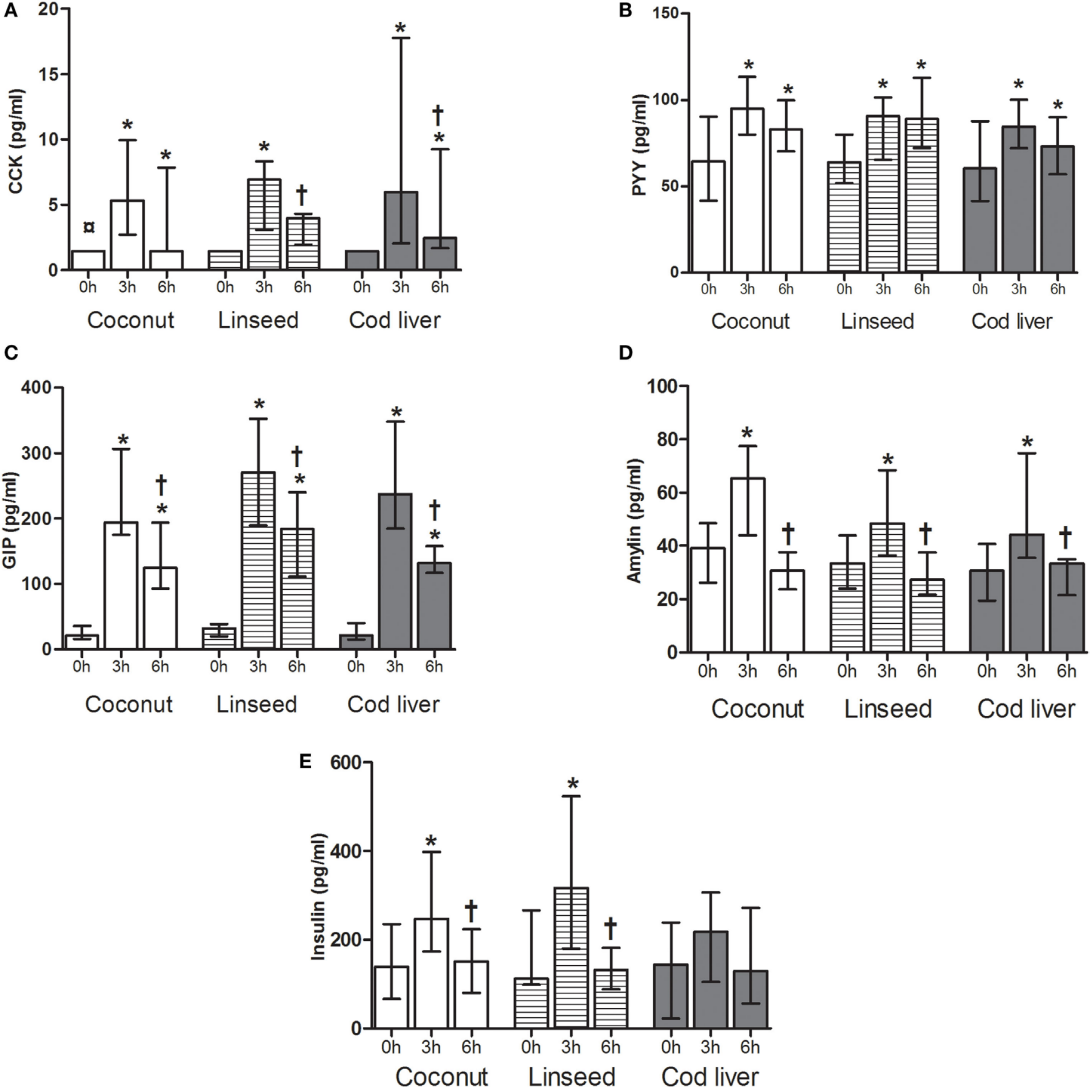
**Plasma levels of CCK (A), PYY (B), GIP (C), amylin (D), and insulin (E) at 0, and 3 and 6 h after the consumption of three test meals enriched with coconut oil [*n* = 14 for PYY, GIP, insulin, and CCK (3 h), *n* = 13 for CCK (0 and 6 h), *n* = 8 for amylin], linseed oil [*n* = 13 for PYY, GIP, insulin (0 and 3 h), and CCK (0 and 3 h), *n* = 12 for insulin (6 h) and CCK (6 h), *n* = 8 for amylin], and cod liver oil [*n* = 12 for PYY, GIP, insulin (0 and 3 h), and CCK, *n* = 11 for insulin (6 h), *n* = 8 for amylin]**. *Median values are significant different from that for 0 h, *P* < 0.05. ^†^Median values are significant different from that for 3 h, *P* < 0.05. ^¤^Baseline is significant different from that for linseed and cod liver *P* < 0.05. Data are shown as median (interquartile range: 25–75th percentiles).

Plasma levels of CCK increased 3 h after intake of all three test meals compared with fasting levels and 6 h after intake in the coconut group and cod liver group whereas plasma levels of GIP and PYY were increased both at 3 and 6 h after intake of all three test meals compared with the respective baseline values (Figures 1A–C). Plasma levels of GIP declined at 6 h compared with the corresponding 3 h for each of the three test meals, whereas CCK declined at 6 h compared with 3 h after intake of the linseed and cod liver meal (Figures 1A,C). However, there were no significant differences in the postprandial change of CCK, PYY, and GIP between the test meals at any time point (Figures 1A–C). There was no difference in baseline values of any of the peptides except for plasma levels of CCK, which at baseline in the coconut group differed significantly from the respective values in the linseed and cod liver group (Figures 1A–E).

To further investigate the postprandial response of a high-fat meal, plasma levels of amylin and insulin were measured. We observed a significant increase in plasma amylin levels from baseline to corresponding 3 h, which declined 6 h after intake of all the test meals, respectively (Figure 1D). Plasma insulin levels increased significantly from baseline to 3 h and then declined 6 h after intake of both coconut and linseed cakes. However, no significant differences in insulin levels were observed after intake of the cod liver cake (Figure 1E). Postprandial amylin and insulin levels were not different between the three different test meals (Figures 1D,E).

### Effects of the Different Test Meals on Adipokine Levels

Adipokines, in particular leptin, have been shown to be involved in the regulation of gut peptides, and we, therefore, investigated the postprandial effects after intake of the three high-fat test meals on serum levels of leptin, adiponectin, resistin, and NAMPT.

**Figure 2:**
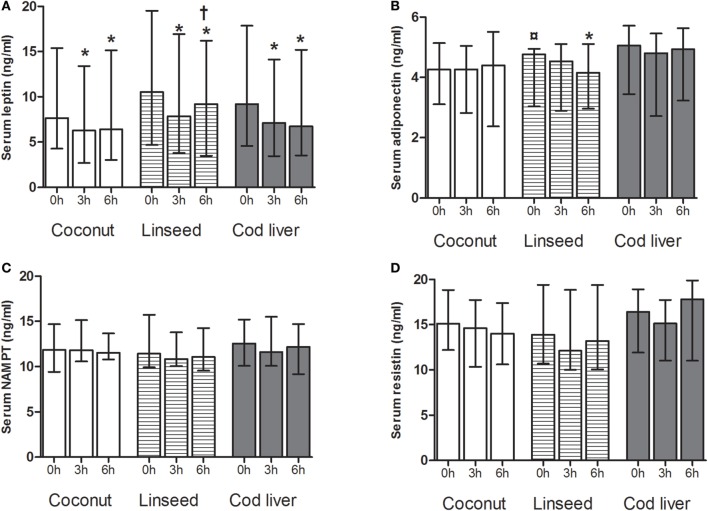
**Serum levels of leptin (A), adiponectin (B), NAMPT (C), and resistin (D) at 0, and 3 and 6 h after the consumption of three test meals enriched with coconut oil (*n* = 14 for all), linseed oil [*n* = 13 for adiponectin, resistin, NAMPT (0 and 3 h), and leptin (0 and 6 h), *n* = 12 for leptin (3 h), NAMPT (6 h)], and cod liver oil [*n* = 12 for leptin, adiponectin, NAMPT, and resistin (0 and 3 h), *n* = 11 for resistin (6 h)]**. *Median values are significant different from that for 0 h, *P* < 0.05. ^†^Median values are significant different from that for 3 h, *P* < 0.05. ^¤^Baseline is significant different from that for coconut and cod liver *P* < 0.05. Data are shown as median (interquartile range: 25–75th percentiles).

Serum levels of leptin decreased significantly at 3 and 6 h after intake of all the three test meals compared with respective baseline values (Figure 2A). Furthermore, serum levels of leptin increased at 6 h after intake of the linseed meal compared with the corresponding 3 h (Figure 2A). Serum adiponectin, NAMPT, and resistin levels did not change after any of the meals except for adiponectin, which declined 6 h after intake of the linseed meal (Figures 2B–D). There was no baseline change in any of the adipokines except for baseline serum adiponectin levels in the linseed group (Figures 2A–D).

### Effects on mRNA Levels of Adipokine-Related Genes in PBMCs

**Figure 3:**
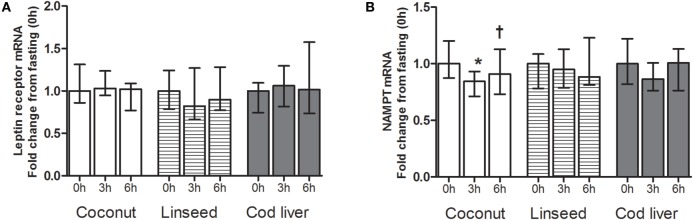
**mRNA levels of leptin receptor (A) and NAMPT (B) in PBMCs at 0 h, and 3 and 6 h after the consumption of three test meals enriched with coconut oil (*n* = 12 for NAMPT and *n* = 7 for leptin receptor), linseed oil [*n* = 11 for NAMPT (0 and 6 h), *n* = 10 for NAMPT (3 h), *n* = 7 for leptin receptor (0 and 6 h), and *n* = 6 for leptin receptor (3 h)], and cod liver oil (*n* = 10 for NAMPT and *n* = 7 for leptin receptor)**. *Median values are significant different from that for 0 h, *P* < 0.05. ^†^Median values are significant different from that for 3 h, *P* < 0.05. Data are shown as median (interquartile range: 25–75th percentiles).

Circulating PBMCs may alter their gene expression as a response to an acute change in the environment (33). We analyzed the gene expression levels of leptin receptor and NAMPT in PBMCs. There were no differences in the mRNA levels in neither leptin receptor nor NAMPT at any time point between the three test meals (Figures 3A,B). We found no postprandial effects in the mRNA level of neither leptin receptor nor NAMPT, except for a significant decline in NAMPT at 3 h compared with baseline after intake of the coconut cake (Figures 3A,B). This postprandial decline increased at 6 h compared with 3 h after meal intake (Figure 3B).

## Discussion

In healthy, normal weight, and young females, a single high-fat meal exerts significant postprandial effects on plasma levels of CCK, PYY, GIP, amylin, and insulin, which increased while leptin decreased. These findings were independent of the fat composition in the single high-fat meal. No postprandial effects were observed in plasma levels of NAMPT and resistin. Furthermore, we observed a postprandial effect in the mRNA level of NAMPT, but not for leptin receptor in PBMCs. The difference in the amount of FAs in the three different cakes might have contributed to the variation seen in the different gastrointestinal peptide responses especially [CCK; Ref. (34)] or adipokines (leptin).

High-fat intake has been shown to induce long-lasting effect on CCK release (35, 36). Accordingly, we observed an increase in the release of CCK, which was elevated up to 6 h after intake. With regard to fat quality, release of CCK has been shown to be stimulated after intake of meals rich in linoleate, whereas meals containing medium-chain triacylglycerols inhibited CCK release (37). Furthermore, Robertson and coworkers reported that the CCK response after a high-fat n-3 meal was significantly delayed compared with intake of saturated fat (35). In the present study, however, we found no difference in the postprandial release of CCK after intake of a single high-fat meal enriched with coconut oil, linseed oil, or cod liver oil. In accordance with our results, no difference was observed in postprandial CCK levels after intake of meals enriched with olive oil or sunflower oil (37). The lack of response could be due to the amount of fats given in the test cakes. The amounts were chosen to have an edible cake.

During fasting, the circulating levels of GIP are low; however, GIP is released in response to ingestion of nutrients. The release of GIP is dependent on the size of the meal, and ingestion of large meals has been shown to mediate secretion of higher amounts of GIP when compared with smaller meals. The major stimuli of GIP release are dietary fat and proteins. Olive oil has been shown to induce GIP to a larger extent than butter (11, 37); however, others found no difference in the GIP release after ingestion of a meal rich in SFA, n-6 FAs, or marine FAs (38). In our study, we observed no difference in the release of GIP induced after intake of a high-fat meal rich in SFA or meals rich in vegetable n-3 or a combination of vegetable and marine n-3 FAs. Previously, GIP has been linked to adipose tissue. GIP induced mRNA expression of inflammatory markers such as interleukin (IL)-6 and IL-1β in human adipocytes (23).

The release of PYY is induced shortly after food intake and decrease in a fasting state. Although all macronutrients induce PYY release, intake of fat has been shown to elicit a larger PYY response compared with both intake of protein and carbohydrate (12). Different types of fat have also been shown to induce release differently with medium-chain length FAs inducing little or no response (14). Plasma PYY was higher after intake of an oleic acid-enriched meal than after a linoleic acid-enriched meal (16). We found no difference in the response of PYY elicited by a high-fat meal enriched with vegetable or a combination of vegetable and marine n-3 FAs compared with a high-fat meal enriched with saturated fat. To our knowledge, few, if any study has previously investigated the effect of intake of meals rich in n-3 FAs on PYY release.

Amylin is an anorexigenic peptide released by the pancreatic β-cells, regulated in a manner similar to insulin. Poppitt and coworkers showed that the intake of a high-fat meal rich in saturated fat or with an improved saturated:unsaturated fatty acid ratio had no effect on postprandial plasma amylin levels (39). We observe a postprandial increase in amylin levels after ingestion of a high-fat meal. This postprandial response did not seem to be affected by the FA composition of the meal. Furthermore, plasma insulin levels were not elevated postprandially by the cod liver cake but only by the linseed and coconut group, which may imply a different response of vegetable compared with marine n-3 FAs. This is in accordance with our previously reported postprandial effects on plasma glucose levels (29) and may potentially indicate a favorable effect of marine compared with vegetable n-3 FAs. Delarue and coworkers have previously shown a decreasing effect of fish oil capsules compared with non-fish oil capsules on insulin response after oral glucose tolerance test in subjects of both genders with type 2 diabetes aged 40–75 (40). Leptin reduces food intake and increase energy expenditure (20), and fasting and re-feeding elicits a decline and a rise in serum leptin, respectively (41, 42). We found that levels of leptin decreased postprandially after intake of a high-fat meal independent of FA composition. Previously, it was shown that a carbohydrate meal induced higher postprandial leptin levels than an isoenergetic fat meal (43). Our results and earlier studies show a decrease in leptin levels after a high-fat meal (44, 45), which was not affected by the saturation of fat in the meal (44).

Ingestion of a high-fat meal or carbohydrate-rich meal reduces postprandial levels of adiponectin (20, 46). Derosa and coworkers reported that adiponectin levels were reduced to 6 and 9 h after an oral fat load (47). In our study, we found no effect on adiponectin levels, 3 h after the intake; however, 6 h after intake of the test meal enriched with linseed oil, we observed a decrease in serum adiponectin levels. Since adiponectin is released from the adipose tissue, our follow-up time of 3 and 6 h after the ingestion of the high-fat meal may be too short to observe effects on circulating adiponectin levels.

A link between gut peptides and adipose tissue has previously been described. Unniappan and coworkers suggested a prolonging effect of leptin on PYY (48). Furthermore, deficiency of leptin has been shown to impair the satiety response of CCK (49), suggesting that gastric leptin also may be involved in early CCK-mediated effects activated by food intake, further underscoring this potential network. We found no indication that intake of meals enriched with marine or vegetable n-3 FAs modulates gastrointestinal peptide and adipokine secretion differently than intake of meals enriched with saturated fat.

The limitations of the present study are a relatively low number of participants and few time points for the postprandial blood samples and the relative small difference in n-3 fatty acid content between the linseed and cod liver group. The amounts were chosen to make the cake edible. Only lean females, students of Akershus University College, Oslo, participated in the study making the extrapolation of the results to other subjects (male, obese) limited. The postprandial response of CCK, PYY, and ghrelin to a high-fat meal seems to be similar in obese and lean male subjects (50). Gender differences are so far not sufficiently addressed and would therefore need to be investigated in further studies. The strength of the study is the controlled study design with a homogenous study population and excellent compliance of the participants. Each subject consumed three test meals and served as their own reference.

In conclusion, the present study shows that, in healthy, young, and lean females, an intake of a high-fat meal enriched with n-3 FAs from different origin elicits the same postprandial response in plasma levels of intestinal peptides with minor effects on adipokine levels compared with intake of a meal enriched with saturated fat.

## Author Contributions

KH and SU designed and together with IN, MM, TD, and BH executed the study. K-HH and TK analyzed the gastrointestinal peptides and adipokines. All authors, have made substantial, direct and intellectual contribution to the work, and approved it for publication.

## Conflict of Interest Statement

The authors declare that the research was conducted in the absence of any commercial or financial relationships that could be construed as a potential conflict of interest.

## Abbreviations

ALA, α-linolenic acid; CCK, cholecystokinin; Ct, cycle threshold; DHA, docosahexaenoic acid; ELISA, enzyme-linked immunosorbant assays; EPA, eicosapentaenoic acid; FAs, fatty acids; GIP, glucose-dependent insulinotropic polypeptide; GUS β, glucuronidase β; IL, interleukin; mRNA, messenger ribonucleic acid; n-3, Omega-3; NAMPT, nicotinamid phosphoribosyl transferase; PBMCs, peripheral blood mononuclear cells; PUFA, polyunsaturated fatty acids; PYY, peptide YY, RIA, radioimmunoassay; RT-qPCR, reverse transcription quantitative polymerase chain reaction; SFA, saturated fatty acids; TBP, TATA box-binding protein; TFA, trifluoroacetic acid.
